# Editorial: Novel mechanism and strategies to overcome relapse after allogeneic stem cell transplantation

**DOI:** 10.3389/fimmu.2023.1248270

**Published:** 2023-07-11

**Authors:** Yao Sun, Xiaohui Zhang, Liangding Hu, Muhammad Bilal Abid

**Affiliations:** ^1^ Senior Department of Hematology, The Fifth Medical Center of People's Liberation Army (PLA) General Hospital, Beijing, China; ^2^ Beijing Key Laboratory of Hematopoietic Stem Cell Transplantation, National Clinical Research Center for Hematologic Disease, Peking University People’s Hospital, Peking University Institute of Hematology, Beijing, China; ^3^ Divisions of Hematology/Oncology & Infectious Diseases, BMT and Cellular Therapy Program, Department of Medicine, Medical College of Wisconsin, Milwaukee, WI, United States

**Keywords:** allogeneic stem cell transplant, acute leukemia, chimeric antigen receptor, IFN-a, lenalidomide, graft-versus-host-disease

The curative potential of allogeneic hematopoietic stem cell transplant (allo-HCT) for hematologic malignancies and nonmalignant conditions was first explored during the 1950s (1). Through persistent clinical investigation and a robust comprehension of transplant immunology, allo-HCT has evolved into a sophisticated technology that offers cure and prolongs survival in patients with hematologic conditions. Major reasons of transplant failure include toxicity due to transplant procedure itself (non-relapse mortality [NRM]) – defined as death while the patient is in disease remission, graft-versus-host disease (GVHD), and relapse of the underlying disease. Longitudinal trend analyses in more than 300,000 patients reported to the Center for International Blood and Marrow Transplantation Research (CIBMTR) demonstrated a decline in NRM and GVHD over the last 4 decades but an increase in relapse. The increasing relapse rates have sustained when adjustments were made due to confounding caused by conditioning intensity and age groups. Leukemia relapse remains the primary cause of treatment failure after allo-HCT and continues to account for at least half of the deaths among recipients of transplant (2). In contemporary era, advancements in acute leukemia genomic and epigenomic profiling have enabled a more thorough understanding of leukemogenesis, resulting in the development of novel targeted and immunologic therapies. The advent of sensitive detection technology has facilitated treatment decisions guided by measurable residual disease (MRD). While novel immunotherapies and T-cell engagement platforms, such as blinatumomab, inotuzumab ozogamicin, and chimeric antigen receptor (CAR) T-cells have shown highly encouraging outcomes patients with acute lymphoblastic leukemia (ALL), relapse continues to pose a significant clinical obstacle and enhancing efficacy through combination strategies remains an area for further exploration (3–5).

The review conducted from the American University of Beirut Medical Center examined innovative strategies for preventing and managing relapse following allo-HCT in patients with ALL. The group showed that the use of maintenance tyrosine kinase inhibitors (TKIs) after allo-HCT in patients with Philadelphia chromosome (Ph+) ALL in first complete remission (CR1) prolongs survival and reduces the risk of relapse, as recommended by the consensus statement from the European Society for Blood and Marrow Transplantation (EBMT). While donor lymphocyte infusion (DLI) has a limited remission rate of no more than 15% in patients with B-cell ALL (B-ALL), prophylactic or pre-emptive DLI may play a role in preventing leukemia relapse, involving multiple escalated doses guided by the occurrence of GVHD. Although CAR-T therapy for B-ALL achieves an early CR rate of nearly 70-80%, more than half of the patients progress within 1 year. Novel sequencing of CAR-T and allo-HCT to reduce relapse rates are being explored. In this regard, a large CIBMTR analysis showed that consolidation allo-HCT after CAR-T results in significant reduction in leukemia relapse with a trend towards better leukemia-free survival (LFS) in 62 patients with R/R B-ALL (6). Limited small-sample studies analyzed the long-term survival of CAR-T followed by second allo-HCT for B-ALL (7). Analysis at the Hebei Yanda Lu Daopei Hospital, provided the largest real-world retrospective study to analyze the outcome of second transplantation used for consolidation after CAR-T in patients who relapsed after the first transplantation. The results of this study indicate that CAR-T therapy, followed by consolidation second transplant, can significantly improve the long-term survival of patients with B-ALL who have experienced relapse following the initial allo-HCT. This study showed that the second transplantation should be conducted within 90 days after CAR-T due to its association with superior LFS and OS, as well as inferior NRM in multivariate analysis. Venetoclax and daratumumab demonstrate promise in the setting of high-risk or relapsed/refractory (R/R) T-cell ALL, an aggressive disease with a dismal outcome.

Currently, there exists no sufficient evidence to support the utilization of alternative agents for post-transplant maintenance in AML, except for patients with FMS-like tyrosine kinase 3 internal tandem duplication mutations (FLT3-ITD) (8). In a study conducted at the Xinqiao Hospital, the investigators showed the potential of azacitidine combined with low-dose lenalidomide as maintainence therapy for patients with AML following allo-HCT. Eligible participants included patients with R/R AML who achieved CR or partial remission prior to allo-HCT, maintained CR with MRD negativity at 100 days post-HCT, and attained hematopoietic reconstitution without grade > 2 GVHD. The results showed that azacitidine combined with low-dose lenalidomide was a safe and effective option for maintenance therapy after allo-HCT in patients with AML and warranted further investigation in a larger cohort with longer follow-up. In another study from the Peking University People’s Hospital, the authors reported real-world data from 4 hospitals about preemptive Interferon (IFN)-α for treating patients with AML with MRD+ disease after allo-HCT. After undergoing allo-HCT, 76.9% of 247 patients received preemptive IFN-a treatment and subsequently attained a MRD negative state. This single-arm retrospective study presents encouraging results which need validation via a prospective randomized controlled trial.

A systematic review and meta-analysis demonstrated the efficacy and safety of vedolizumab, a monoclonal antibody that impedes the interaction between α4β7 integrins on T-lymphocytes and MAdCAM1 on gut endothelial cells, for the treatment and prevention of gastrointestinal acute GVHD (GI-aGVHD). The study found that patients receiving vedolizumab for treatment exhibited a higher response rate and lower incidence of adverse events during the early stages of GI-aGVHD.

Besides preemptive and maintenance treatment following allo-HCT, optimizing donor selection in a risk-adapted manner can also mitigate the relapse risk. Two large CIBMTR analyses demonstrated a noteworthy decrease in relapse rates among adult patients with B-ALL and AML who underwent allo-HCT with younger matched unrelated donors (MUD), as opposed to older matched sibling donors (9, 10). The results highlight that younger MUD donor type exerts a stronger graft-versus-leukemia effect and should be preferred in patients at a particularly higher risk for relapse after allo-HCT.

This Research Topic offers a valuable forum for advancing novel approaches and mechanisms to address relapse following allo-HCT.

## Author contributions

YS and LH: study concept, design, and drafted the manuscript. MBA provided expert input. All authors contributed to the article and approved the submitted version.

## References

[1] Copelan EA. Hematopoietic stem-cell transplantation. N Engl J Med (2006) 354(17):1813–26. doi: 10.1056/NEJMra052638 16641398

[2] Phelan R Chen M Bupp C Bolon YT Broglie L Brunner-Grady J. Updated trends in hematopoietic cell transplantation in the united states with an additional focus on adolescent and young adult transplantation activity and outcomes. Transplant Cell Ther (2022) 28(7):409.e1–.e10. doi: 10.1016/j.jtct.2022.04.012 PMC9840526 35447374

[3] Hua J Zhang J Zhang X Wu X Zhou L Bao X. Donor-derived anti-Cd19 car T cells compared with donor lymphocyte infusion for recurrent b-all after allogeneic hematopoietic stem cell transplantation. Bone Marrow Transplant (2021) 56(5):1056–64. doi: 10.1038/s41409-020-01140-6 33235353

[4] Stein AS Kantarjian H Gökbuget N Bargou R Litzow MR Rambaldi A. Blinatumomab for acute lymphoblastic leukemia relapse after allogeneic hematopoietic stem cell transplantation. Biol Blood Marrow Transplant (2019) 25(8):1498–504. doi: 10.1016/j.bbmt.2019.04.010 31002989

[5] Papayannidis C Sartor C Dominietto A Zappone E Arpinati M Marconi G. Inotuzumab ozogamicin and donor lymphocyte infusion is a safe and promising combination in relapsed acute lymphoblastic leukemia after allogeneic stem cell transplant. Hematol Oncol (2021) 39(4):580–3. doi: 10.1002/hon.2886 33963566

[6] Park JH Nikiforow S Kim S Hu Z-H Moskop A Ahmed S. Impact of allogeneic hematopoietic cell transplantation (Hct) as consolidation following Cd19 chimeric antigen receptor (Car) T cell therapy for treatment of relapsed acute lymphoblastic leukemia (All). Blood (2021) 138(Supplement 1):3880–. doi: 10.1182/blood-2021-148652

[7] Chen YH Zhang X Cheng YF Chen H Mo XD Yan CH. Long-term follow-up of Cd19 chimeric antigen receptor T-cell therapy for Relapsed/Refractory acute lymphoblastic leukemia after allogeneic hematopoietic stem cell transplantation. Cytotherapy (2020) 22(12):755–61. doi: 10.1016/j.jcyt.2020.08.002 32861622

[8] Döhner H Wei AH Appelbaum FR Craddock C DiNardo CD Dombret H. Diagnosis and management of aml in adults: 2022 recommendations from an international expert panel on behalf of the eln. Blood (2022) 140(12):1345–77. doi: 10.1182/blood.2022016867 35797463

[9] Abid MB Estrada-Merly N Zhang M-J Chen K Bredeson C Allan D. Younger matched unrelated donors confer a decreased relapse risk as compared to older sibling donors for adult b-cell all patients undergoing allogeneic hematopoietic cell transplantation. Transplant Cell Therapy Off Publ Am Soc Transplant Cell Ther (2023) 29(2):S24–S5. doi: 10.1016/S2666-6367(23)00097-0 PMC10592336 37481243

[10] Abid MB Estrada-Merly N Zhang M-J Chen K Allan D Bredeson C. Impact of donor age on allogeneic hematopoietic cell transplantation outcomes in older adults with acute myeloid leukemia. Transplant Cell Ther (2023) S2666-6367(23):01380–5. doi: 10.1016/j.jtct.2023.06.020 PMC10528825 37406882

